# Elongation factor-P at the crossroads of the host-endosymbiont interface

**DOI:** 10.15698/mic2015.10.232

**Published:** 2015-09-23

**Authors:** Andrei Rajkovic, Anne Witzky, William Navarre, Andrew J. Darwin, Michael Ibba

**Affiliations:** 1 Molecular, Cellular and Developmental Biology Program, Ohio State University, Columbus, Ohio, USA.; 2 Department of Molecular Genetics, Ohio State University, Columbus, Ohio, USA.; 3 Department of Molecular Genetics, University of Toronto, Toronto, Ontario, Canada.; 4 Department of Microbiology, New York University School of Medicine, New York, New York, USA.; 5 Department of Microbiology and Center for RNA Biology, Ohio State University, Columbus, Ohio, USA.

**Keywords:** elongation factor-P, rhamnosylation, modification, translation

## Abstract

Elongation factor P (EF-P) is an ancient bacterial translational factor that aids the ribosome in polymerizing oligo-prolines. EF-P structurally resembles tRNA and binds in-between the exit and peptidyl sites of the ribosome to accelerate the intrinsically slow reaction of peptidyl-prolyl bond formation. Recent studies have identified in separate organisms, two evolutionarily convergent EF-P post-translational modification systems (EPMS), split predominantly between gammaproteobacteria, and betaproteobacteria. In both cases EF-P receives a post-translational modification, critical for its function, on a highly conserved residue that protrudes into the peptidyl-transfer center of the ribosome. EPMSs are comprised of a gene(s) that synthesizes the precursor molecule used in modifying EF-P, and a gene(s) encoding an enzyme that reacts with the precursor molecule to catalyze covalent attachment to EF-P. However, not all organisms genetically encode a complete EPMS. For instance, some symbiotic bacteria harbor efp and the corresponding gene that enzymatically attaches the modification, but lack the ability to synthesize the substrate used in the modification reaction. Here we highlight the recent discoveries made regarding EPMSs, with a focus on how these incomplete modification pathways shape or have been shaped by the endosymbiont-host relationship.

For the majority of gammaproteobacteria the EF-P modification pathway consists of three genes, two of which are paralogs usually found adjacent to *efp *1,2. The 2,3- lysine aminomutase paralog YjeK synthesizes the non-proteinogenic amino acid (R)-β-lysine, which serves as the substrate for the lysyl-tRNA synthetase paralog PoxA. PoxA activates and then ligates (R)-β-lysine onto K34 (*Escherichia coli *K-12 numbering) of EF-P to form an intermediate post-translationally modified state that gets further hydroxylated on the modified lysine residue, K34, by the hydroxylase YfcM. The attachment of β-lysine has been demonstrated to enhance the apparent affinity (*K*_m_) of EF-P for the ribosome, while the role of the hydroxylation remains entirely unknown. Absence of the β-lysine modification renders *Salmonella enterica* avirulent, as a number of the genes involved with pathogenicity encode poly-proline stretches. There are, however, gammaproteobacteria that exist as endosymbionts and lack genes with homology to *yfcM *and *yjeK*, yet still have *poxA* and *efp*. One such organism is *Buchnera aphidicola* str. APS, which has evolved to be an obligate endosymbiont and provide its host *Acyrthosiphon pisum aphid* with essential amino acids that are usually scare in a phloem-based diet 3. Lysine is one of the ten essential amino acids that are not synthesized by *A. pisum*, and therefore the absence of *yjeK *from *B. aphidicola* str. APS prevents the conversion of L-lysine to (R)-β-lysine, which can then in turn be used to supply *A. pisum* with more lysine.

Due to the considerable portion of the ancestral genes that have been shed from *B. aphidicola* str. APS, only 4 genes have stretches of three or more prolines, including 1 gene involved in the biosynthesis of the essential amino acid, histidine. By decreasing the number of proteins that are dependent on EF-P for translation, the necessity to modify EF-P post-translationally may in effect be eliminated. However, other species of *B. aphidicola* appear to still encode YjeK*, *PoxA* and *EF-P, yet also only have between 4-5 genes with poly-prolines. With evidence suggesting *B. aphidicola* mutate at a rapid rate compared to other free-living bacteria, it is possible these other *B. aphidicola *species will eventually lose *yjeK *4. Nevertheless, EF-P could remain modified in *Buchnera aphidicola* str. APS, but instead with α-lysine, although it is a less favorable substrate for PoxA.

A vastly different EF-P modification pathway with similar physiological importance was recently discovered in the related betaproteobacterium, *Pseudomonas aeruginosa*. *P. aeruginosa* expresses the glycosyltransferase EarP that binds the sugar nucleotide dTDP-L-rhamnose, and attaches a cyclic L-rhamnose onto the conserved R32 residue of EF-P, analogous to K34 of *E. coli *5,6. Though rhamnose deviates chemically and geometrically from (R)-β-lysine, the glycan moiety remains important to the function of EF-P. However, unlike the EF-P modification pathway in *E. coli *and *S. enterica*, EarP in *P. aeruginosa* modifies EF-P with a substrate that has multiple physiological roles. dTDP-L-rhamnose is formed from a conserved sugar nucleotide synthesis pathway encoded by four genes in the *rmlABCD* operon and primarily provides bacteria with a substrate for glycosylating the lipopolysaccharide (LPS) and flagella. Despite the broad conservation of the RmlABCD pathway, not all organisms with *earP* and *efp* encode the proteins required to synthesize dTDP-L-rhamnose. One such organism is *Micavibrio aeruginosavorus*, an epibiotic alphaproteobacteria surviving on the surface of bacteria like *P. aeruginosa*.

*M. aeruginosavorus* is an obligate predator that alternates between two distinct life cycles: a metabolically quiescent attack phase and a metabolically active growth phase 7. During the attack phase, *M. aeruginosavorus* mainly expresses proteins involved in motility and chemotaxis to seek out gram-negative bacteria for prey. Once a suitable host is detected, *M. aeruginosavorus* attaches onto, and most likely compromises the integrity of the outer membrane to allow for the extraction of nutrients. Upon attachment a burst of transcriptional activity is observed in *M. aeruginosavorus* for a variety of genes including *efp *and *earP*. However, *M. aeruginosavorus *does not encode the RmlABCD pathway, calling into question whether EF-P is modified.

In contrast to *B. aphidicola*, *M. aeruginosavorus *encodes 128 genes with stretches of three or more continuous prolines, the majority of which are strictly transcribed during the growth phase, suggesting EF-P is important to facilitate the transition from attack phase to growth phase. While the importance of EF-P may be clear in *M. aeruginosavorus*, the certitude of a modified EF-P is harder to claim, as the threshold at which poly-proline induced pausing demands EF-P to be modified is unknown. However, it is not inconceivable to hypothesize that when the host’s outer membrane is breeched, an ample source of exogenous dTDP-L-rhamnose making its way to be added to the LPS is instead hijacked by *M. aeruginosavorus* to allow for EF-P to be modified (Fig 1). Consequently, if *M. aeruginosavorus* required EF-P to be modified in order to prevent widespread pausing when translating a poly-proline rich proteome, the host-range would be restricted to organisms that can synthesize dTDP-L-rhamnose. Nevertheless, over 1000 bacterial organisms encode the genes to synthesize dTDP-L-rhamnose, signifying the potentially broad therapeutic value of *M. aeruginosavorus* as a live antibiotic to control infections.

**Figure 1:**
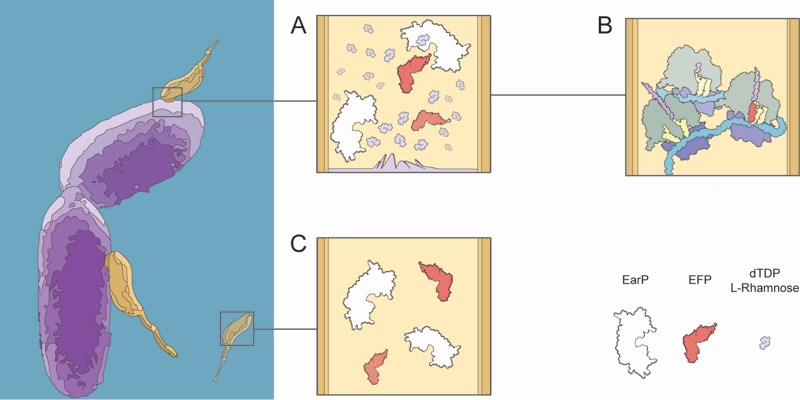
FIGURE 1: The role of EF-P in *M. aeruginosavorus* during host invasion. The far left panel depicts *P. aeruginosa* drawn in purple and *M. aeruginosavorus* in yellow. **(A)** The image illustrates a breach in the outer membrane allowing for dTDP-L-rhamnose to diffuse into *M. aeruginosavorus *where EF-P (red) is then modified by EarP (white). **(B) **As a result of EF-P being modified ribosomes (grey and purple) can effectively translate mRNA (blue) with a poly-proline stretch. **(C) **On the other hand during the attack phase EF-P is unmodified and therefore poly-proline translation is absent.

Even though EF-P is encoded in all bacterial genomes that have been sequenced, it is clear the EF-P modification pathway is neither conserved nor complete amongst bacteria. So far, the only partial EPMSs to have been identified are in the bacterial endosymbionts, *M. aeruginosavorus* and *B. aphidicola*. Both endosymbionts are deficient for the genes required to synthesize the modified substrate. However, the absence of these genes may have different physiological consequences for the host-endosymbiont dynamic. While the exoparasite *M. aeruginosavorus* seems to require a host that can provide the substrate for modifying EF-P, *B. aphidicola* appears to have lost the ability to modify EF-P in order to compensate the metabolically limited aphid host with a sufficient supply of the essential amino acid lysine. As more EF-P modification pathways are uncovered, future endeavors will determine whether an evolutionary trend of partial EPMSs exists for obligate symbionts.
